# Supplementary material for: Parasite infection disrupts escape behaviours in fish shoals
Source: Proc Biol Sci. 2020 Nov 4;287(1938):20201158. doi: 10.1098/rspb.2020.1158 (PMC7735259; doi:10.1098/rspb.2020.1158)
Supplement: Statistical summary and R script analysis and graphs [file rspb20201158supp1.pdf]

Article title: Parasite infection disrupts escape behaviours in fish shoals
Journal name: Proceedings of the Royal Society B
Author names: Nicolle Demandt[1], Marit Praetz[1], Ralf, H.J.M. Kurvers[2], Jens Krause[3,4], Joachim Kurtz[1] and Jörn P. Scharsack[1].
Affiliations:
[1]Institute for Evolution and Biodiversity, University of Münster, Hüfferstrasse 1, 48149, Germany
[2]Center for Adaptive Rationality, Max Planck Institute for Human Development, Müggelseedamm 310, Berlin, Germany
[3]Leibniz-Insitute of Freshwater Ecology and Inland Fishery, Müggelseedamm 310, Berlin, Germany
[4]Faculty of Life Sciences Humboldt-Universität zu Berlin, Invalidenstrasse 42, 10115 Berlin, Germany
Article DOI: 10.1098/rspb.2020.1158
DOI dataset: 10.5061/dryad.5dv41ns46
Email corresponding author: n_vand10@uni-muenster.de

Table S1. (generalized) linear mixed models to analyse the influence of treatment and group on the A) escape zone and B) Fleeing depth of each individual. Significant p-values are highlighted in bold.

	**A) Escape zone**			**B) Fleeing depth**		
	Df	LRT	p-value	Df	LRT	p-value
Length (centred)	1	3.9773	**0.046**	1	2.3837	0.123
Treatment	1	4.4799	**0.034**	1	8.2689	**0.004**
Subgroup	2	9.2149	**0.010**	2	13.407	**0.001**
Treatment x Subgroup	2	7.1646	**0.028**	2	13.712	**0.001**

Table S2. Summary table of the (generalized) linear mixed models to analyse the influence of treatment and group on the A) escape zone and B) Fleeing depth of each individual.

	**A) Escape zone**			**B) Fleeing depth**		
Fixed effects	Estimate	Std. Error	z value	Estimate	Std. Error	T value
intercept	0.607	0.578	1.051	36.563	2.027	18.036
Treatment Infected	1.765	0.871	2.026	-4.392	2.617	-1.678
Subgroup Stimuli	-0.236	0.655	-0.360	2.629	2.225	1.182
Subgroup Transmission	-0.263	0.647	-0.406	2.666	2.225	1.198
Length (centred)	-0.285	0.134	-2.137	NA	NA	NA
Treatment Infected x Subgroup Stimuli	-1.821	0.957	-1.902	2.499	2.865	0.872
Treatment Infected x Subgroup Transmission	0.838	1.105	0.758	-8.726	2.866	-3.045
Random effects	Variance	Std. Dev.		Variance	Std. Dev.	
Group ID: Trial no	1.076	1.038		9.689	3.113	
Trial no	0.999	1.000		13.075	3.616	
Residual	NA	NA		40.458	6.361	

Table S3. Post hoc tests to determine which specific groups statistically differed in their A) escape zone and B) Fleeing depth. Significant p-values are highlighted in bold.

	**A) Escape zone**		**B) Fleeing depth**	
**Comparisons**	z	p	t	p
**Control:** response vs stimuli group	0.360	1.000	-1.123	0.807
**Control:** response vs transmission group	0.406	1.000	-1.138	0.788
**Control:** stimuli vs transmission group	0.042	1.000	-0.016	1.000
**Infected:** response vs stimuli group	2.955	**0.009**	-2.676	**0.033**
**Infected:** response vs transmission group	-0.641	1.000	3.165	**0.009**
**Infected:** stimuli vs transmission group	-3.239	**0.004**	5.920	**<0.001**
**Response group:** control vs infected	-2.026	**0.043**	1.593	0.119
**Stimuli group:** control vs infected	0.075	0.941	0.704	0.485
**Transmission group:** control vs infected	-2.687	**0.007**	4.755	**<0.001**

Table S4. (generalized) linear mixed models to analyse the influence of treatment and group on the time spent in the dangerous zone. Significant p-values are highlighted in bold.

	**Time spent in dangerous zone**		
	Df	LRT	p-value
Length (centred)	1	8.617	**0.003**
Treatment	1	5.6359	**0.018**
Subgroup	2	1.598	0.450
Bird strike (before or after)	1	14.612	**<0.001**
Treatment x Subgroup	2	4.792	*0.091*
Bird strike (before or after) x Subgroup	2	1.010	0.603
Bird strike (before or after) x Treatment	1	35.707	**<0.001**
Bird strike (before or after) x Treatment x Subgroup	2	1.4952	0.474

Table S5. Summary table of the (generalized) linear mixed models to analyse the time spent in the dangerous zone.

	**Time spent in dangerous zone**		
Fixed effects	Estimate	Std. Error	t value
Intercept	210.213	8.792	23.908
Treatment Infected	54.152	10.818	5.006
Bird strike (before or after) Before	54.156	7.088	7.641
Length (centred)	-1.301	0.430	-3.024
Treatment Infected x Bird strike (before or after) Before	-50.067	8.068	-6.206
Random effects	Std. Dev		
Fish ID: Group ID: Trial no	12.673		
Group ID: Trial no	19.728		
Trial no	15.104		
Residual	28.335		

Table S6. Post hoc tests to determine which specific groups statistically differed in their time spent in the dangerous zone. Significant p-values are highlighted in bold.

	**Time spent in dangerous zone**	
**Comparisons**	t	p
**After bird strike:** Control vs Infected	-5.006	**<0.001**
**Before bird strike:** Control vs Infected	-0.404	0.691
**Control:** After vs before bird strike	-7.641	**<0.001**
**Infected:** After vs before bird strike	-1.061	0.290

Fig. S7

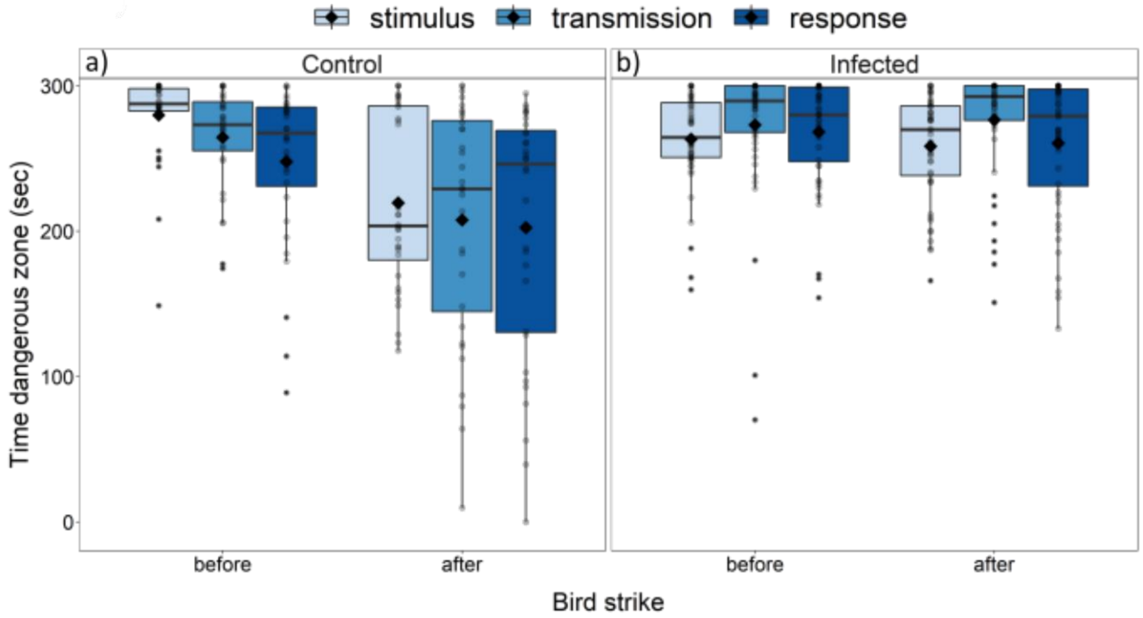

Figure S7: Time spent in the dangerous zone for fish in the a) control and b) infected treatment groups before and after a simulated bird strike. Results are shown per compartment group: stimulus (light blue), transmission (blue) and response (dark blue), split by time (i.e., before versus after the bird strike). Edges of the box plots indicate first and third quartiles, the solid lines the median, diamonds the mean, whiskers the highest and lowest values within 1.5-fold of the inter-quartile range, all (black) dots outside of the whiskers are outliers and the transparent dots represent all individual data points.

Table S8. (generalized) linear mixed models to analyse the influence of parasite load and bird strike (before or after) on A) the fleeing depth and B) the time spent in the dangerous zone. Significant p-values are highlighted in bold.

	**A) Fleeing depth**			**B) Time spent in dangerous zone**		
	Df	LRT	p-value	df	LRT	p-value
Parasite load	1	3.744	*0.053*	1	3.672	*0.055*
Bird strike (before or after)	NA	NA	NA	1	0.086	0.770
Bird strike (before or after) x Parasite load	NA	NA	NA	1	0.732	0.392

Table S9. Summary table of the (generalized) linear mixed models to analyse the influence of parasite load and bird strike (before or after) on A) the fleeing depth and B) the time spent in the dangerous zone.

	**A) Fleeing depth**			**B) Time spent in dangerous zone**		
Fixed effects	Estimate	Std. Error	t value	Estimate	Std. Error	T value
intercept	39.960	7.356	5.432	232.30	23.35	-2.890
Parasite load	-74.642	38.644	-1.932	238.31	120.66	3.195
Random effects	Variance		Std. Dev.	Variance		Std. Dev.
Fish ID: Group ID	NA		NA	186.7		13.66
Group ID	0.000		0.000	460.1		21.45
Residual	66.79		8.173	1165.5		34.14

```
library(readxl)
library(ggplot2)
library(lme4)
library(car)
library(nortest)
library(emmeans)
library(nlme)
library(ggpubr)


str(Marit<-read_excel('D:\\Users\\User\\Documents\\PhD
Munster\\Supervision BSc students\\BSc thesis
2018\\Marit\\Manuscript\\Submission proc
b\\Demandt_et_al_dataset.xlsx',1))
Marit$Treatment<-factor(Marit$Treatment)
Marit$FishID<-factor(Marit$FishID)
Marit$GroupID<-factor(Marit$GroupID)
Marit$TreatmentGroup<-factor(Marit$TreatmentGroup)
Marit$Fleezone<-factor(Marit$Fleezone)
Marit$InfectionStatus<-factor(Marit$InfectionStatus)
Marit$BeforeOrAfter<-factor(Marit$BeforeOrAfter)
Marit$ExpNr<-factor(Marit$ExpNr)

## Make subset of data, so that it only contains the after bird strike
data
Maritafter<-subset(Marit,BeforeOrAfter=="After")



                    ## Fleeing depth analysis!!  ###
# Center lenght fish
L1<-scale(Maritafter$Length,scale=TRUE,center=TRUE)

m0a<-
lmer(Fleeingdepth~Treatment*TreatmentGroup+L1+(1|ExpNr/GroupID),data=Mari
tafter,control=lmerControl(calc.derivs=F))
summary(m0a)
#Linear mixed model fit by REML ['lmerMod']
#Formula: Fleeingdepth ~ Treatment * TreatmentGroup + L1 + (1 |
ExpNr/GroupID)
#   Data: Maritafter
#Control: lmerControl(calc.derivs = F)
#
#REML criterion at convergence: 1611
#
#Scaled residuals:
#    Min      1Q  Median      3Q     Max
#-2.7722 -0.5760  0.0734  0.6163  3.3030
#
#Random effects:
# Groups        Name        Variance Std.Dev.
# GroupID:ExpNr (Intercept) 13.70    3.701
# ExpNr         (Intercept) 13.37    3.656
# Residual                  39.64    6.296
#Number of obs: 240, groups:  GroupID:ExpNr, 61; ExpNr, 20
#
#Fixed effects:
#                                       Estimate Std. Error t value
```

```
#(Intercept)                            36.4845     2.1503  16.967
#TreatmentB                             -4.3610     2.7755  -1.571
#TreatmentGroupStimuli                   2.6532     2.4294   1.092
#TreatmentGroupTransmission              2.7433     2.4299   1.129
#L1                                      0.7353     0.4448   1.653
#TreatmentB:TreatmentGroupStimuli        2.5068     3.1258   0.802
#TreatmentB:TreatmentGroupTransmission  -8.5955     3.1269  -2.749
#
#Correlation of Fixed Effects:
#             (Intr) TrtmnB TrtmGS TrtmGT L1     TB:TGS
#TreatmentB  -0.775
#TrtmntGrpSt -0.565  0.438
#TrtmntGrpTr -0.565  0.438  0.500
#L1          -0.022  0.007  0.006  0.019
#TrtmntB:TGS  0.439 -0.567 -0.777 -0.388  0.019
#TrtmntB:TGT  0.439 -0.567 -0.388 -0.777  0.010  0.508

m1<-update(m0a,REML=F)
drop1(m1,test="C")
#Single term deletions
#
#Model:
#Fleeingdepth ~ Treatment * TreatmentGroup + L1 + (1 | ExpNr/GroupID)
#                         Df    AIC     LRT  Pr(Chi)
#<none>                      1649.1
#L1                        1 1649.5  2.3837 0.122603
#Treatment:TreatmentGroup  2 1657.9 12.8375 0.001631 **
#---
#Signif. codes:  0 '***' 0.001 '**' 0.01 '*' 0.05 '.' 0.1 ' ' 1

m2<-update(m1,~.-L1)
summary(m2)
#Linear mixed model fit by maximum likelihood  ['lmerMod']
#Formula: Fleeingdepth ~ Treatment + TreatmentGroup + (1 | ExpNr/GroupID)
+
#    Treatment:TreatmentGroup
#   Data: Maritafter
#Control: lmerControl(calc.derivs = F)
#
#     AIC      BIC   logLik deviance df.resid
#  1649.4   1680.8   -815.7   1631.4      231
#
#Scaled residuals:
#    Min      1Q  Median      3Q     Max
#-2.7316 -0.6362  0.0885  0.6151  3.4668
#
#Random effects:
# Groups        Name        Variance Std.Dev.
# GroupID:ExpNr (Intercept)  9.689   3.113
# ExpNr         (Intercept) 13.075   3.616
# Residual                  40.458   6.361
#Number of obs: 240, groups:  GroupID:ExpNr, 61; ExpNr, 20
#
#Fixed effects:
#                                     Estimate Std. Error t value
#(Intercept)                            36.563      2.027  18.036
#TreatmentB                             -4.392      2.617  -1.678
#TreatmentGroupStimuli                   2.629      2.225   1.182
```

```
#TreatmentGroupTransmission                 2.666      2.225   1.198
#TreatmentB:TreatmentGroupStimuli          2.499      2.865   0.872
#TreatmentB:TreatmentGroupTransmission   -8.726      2.866  -3.045
#
#Correlation of Fixed Effects:
#            (Intr) TrtmnB TrtmGS TrtmGT TB:TGS
#TreatmentB  -0.775
#TrtmntGrpSt -0.549  0.425
#TrtmntGrpTr -0.549  0.425  0.500
#TrtmntB:TGS  0.426 -0.550 -0.777 -0.388
#TrtmntB:TGT  0.426 -0.550 -0.388 -0.776  0.505

drop1(m2,test="C")
#Single term deletions
#
#Model:
#Fleeingdepth ~ Treatment + TreatmentGroup + (1 | ExpNr/GroupID) +
#    Treatment:TreatmentGroup
#                         Df    AIC    LRT  Pr(Chi)
#<none>                      1649.5
#Treatment:TreatmentGroup  2 1659.2 13.712 0.001053 **
#---
#Signif. codes:  0 '***' 0.001 '**' 0.01 '*' 0.05 '.' 0.1 ' ' 1

m3<-update(m2,REML=T)

plot(m3)
qqPlot(resid(m3))
ad.test(resid(m3))

#        Anderson-Darling normality test
#
#data:  resid(m4)
#A = 0.61959, p-value = 0.1057

plot(resid(m3)~Maritafter$Treatment)
leveneTest(resid(m3)~Maritafter$Treatment)
#Levene's Test for Homogeneity of Variance (center = median)
#       Df F value  Pr(>F)
#group   1  3.0844 0.08033 .
#      238
#---
#Signif. codes:  0 '***' 0.001 '**' 0.01 '*' 0.05 '.' 0.1 ' ' 1

plot(resid(m3)~Maritafter$TreatmentGroup)
leveneTest(resid(m3)~Maritafter$TreatmentGroup)
#Levene's Test for Homogeneity of Variance (center = median)
#       Df F value Pr(>F)
#group   2  1.1965 0.3041
#      237

emmeans(m3,pairwise~TreatmentGroup|Treatment,simple="each",adjust="Bonfer
roni")$contrast
#$`simple contrasts for TreatmentGroup`
#Treatment = A:
# contrast                  estimate       SE    df t.ratio p.value
# Response - Stimuli      -2.62908835 2.342145 36.11  -1.123  0.8072
# Response - Transmission -2.66606018 2.342145 36.11  -1.138  0.7875
```

```
# Stimuli - Transmission  -0.03697183 2.342145 36.11  -0.016  1.0000
#
#Treatment = B:
# contrast                   estimate       SE    df t.ratio p.value
# Response - Stimuli      -5.08481903 1.900082 37.97  -2.676  0.0328    *
# Response - Transmission  6.01880695 1.901451 37.61   3.165  0.0092
**
# Stimuli - Transmission  11.10362598 1.875690 42.51   5.920  <.0001
***
#
#P value adjustment: bonferroni method for 3 tests
#
#$`simple contrasts for Treatment`
#TreatmentGroup = Response:
# contrast  estimate       SE    df t.ratio p.value
# A - B     4.391623 2.757341 41.02   1.593  0.1189
#
#TreatmentGroup = Stimuli:
# contrast  estimate       SE    df t.ratio p.value
# A - B     1.935892 2.748844 41.12   0.704  0.4852
#
#TreatmentGroup = Transmission:
# contrast  estimate       SE    df t.ratio p.value
# A - B    13.076490 2.749790 41.08   4.755  <.0001     ***


## To gather the p-values of loose variables
 n0<-
lmer(Fleeingdepth~1+(1|ExpNr/GroupID),data=Maritafter,control=lmerControl
(calc.derivs=F),REML=F)
n1<-update(n0,~.+Treatment)
anova(n0,n1)
#Data: Maritafter
#Models:
#n0: Fleeingdepth ~ 1 + (1 | ExpNr/GroupID)
#n1: Fleeingdepth ~ (1 | ExpNr/GroupID) + Treatment
#   npar    AIC    BIC  logLik deviance  Chisq Df Pr(>Chisq)
#n0    4 1674.8 1688.7 -833.41   1666.8
#n1    5 1668.5 1686.0 -829.28   1658.5 8.2689  1   0.004033 **
#---
#Signif. codes:  0 '***' 0.001 '**' 0.01 '*' 0.05 '.' 0.1 ' ' 1

n2<-update(n0,~.+TreatmentGroup)
anova(n0,n2)
#Data: Maritafter
#Models:
#n0: Fleeingdepth ~ 1 + (1 | ExpNr/GroupID)
#n2: Fleeingdepth ~ (1 | ExpNr/GroupID) + TreatmentGroup
#   npar    AIC    BIC  logLik deviance  Chisq Df Pr(>Chisq)
#n0    4 1674.8 1688.7 -833.41   1666.8
#n2    6 1665.4 1686.3 -826.71   1653.4 13.407  2   0.001227 **
#---
#Signif. codes:  0 '***' 0.001 '**' 0.01 '*' 0.05 '.' 0.1 ' ' 1


rm(m0a,m1,m2,m3,n0,n1,n2)
```

```
                    #### Flee zone analysis ###
m0a<-
glmer(Flee~Treatment*TreatmentGroup+L1+(1|ExpNr/GroupID),data=Maritafter,
family=binomial(link="probit"),na.action=na.omit,control=glmerControl(cal
c.derivs=F))    ## Best model
summary(m0a)
#Generalized linear mixed model fit by maximum likelihood (Laplace
Approximation) [glmerMod]
# Family: binomial  ( probit )
#Formula: Flee ~ Treatment * TreatmentGroup + L1 + (1 | ExpNr/GroupID)
#   Data: Maritafter
#Control: glmerControl(calc.derivs = F)
#
#     AIC      BIC   logLik deviance df.resid
#   224.1    255.5   -103.1    206.1      231
#
#Scaled residuals:
#     Min       1Q   Median       3Q      Max
#-2.65096 -0.31734  0.07044  0.29354  1.71466
#
#Random effects:
# Groups        Name        Variance Std.Dev.
# GroupID:ExpNr (Intercept) 1.0764   1.0375
# ExpNr         (Intercept) 0.9992   0.9996
#Number of obs: 240, groups:  GroupID:ExpNr, 61; ExpNr, 20
#
#Fixed effects:
#                                       Estimate Std. Error z value
Pr(>|z|)
#(Intercept)                              0.6070     0.5776   1.051
0.2933
#TreatmentB                               1.7647     0.8710   2.026
0.0428 *
#TreatmentGroupStimuli                   -0.2356     0.6553  -0.360
0.7192
#TreatmentGroupTransmission              -0.2630     0.6472  -0.406
0.6845
#L1                                      -0.2853     0.1335  -2.137
0.0326 *
#TreatmentB:TreatmentGroupStimuli        -1.8212     0.9574  -1.902
0.0571 .
#TreatmentB:TreatmentGroupTransmission   0.8375     1.1051   0.758
0.4485
#---
#Signif. codes:  0 '***' 0.001 '**' 0.01 '*' 0.05 '.' 0.1 ' ' 1
#
#Correlation of Fixed Effects:
#             (Intr) TrtmnB TrtmGS TrtmGT L1     TB:TGS
#TreatmentB  -0.662
#TrtmntGrpSt -0.549  0.366
#TrtmntGrpTr -0.556  0.368  0.490
#L1          -0.020 -0.042 -0.040  0.007
#TrtmntB:TGS  0.375 -0.662 -0.687 -0.335  0.079
#TrtmntB:TGT  0.325 -0.541 -0.288 -0.586  0.016  0.493

drop1(m0a,test="C")
#Single term deletions
#
```

```
#Model:
#Flee ~ Treatment * TreatmentGroup + L1 + (1 | ExpNr/GroupID)
#                          Df    AIC    LRT Pr(Chi)
#<none>                       224.15
#L1                        1 226.12 3.9773 0.04612 *
#Treatment:TreatmentGroup  2 227.31 7.1646 0.02781 *
#---
#Signif. codes:  0 '***' 0.001 '**' 0.01 '*' 0.05 '.' 0.1 ' ' 1



emmeans(m0a,pairwise~TreatmentGroup|Treatment,simple="each",adjust="Bonfe
rroni")$contrast
#$`simple contrasts for TreatmentGroup`
#Treatment = A:
# contrast                   estimate        SE  df z.ratio p.value
# Response - Stimuli       0.23562553 0.6553210 Inf   0.360  1.0000
# Response - Transmission  0.26298017 0.6471927 Inf   0.406  1.0000
# Stimuli - Transmission   0.02735464 0.6575106 Inf   0.042  1.0000
#
#Treatment = B:
# contrast                   estimate        SE  df z.ratio p.value
# Response - Stimuli       2.05678124 0.6961075 Inf   2.955  0.0094
**
# Response - Transmission -0.57449457 0.8958371 Inf  -0.641  1.0000
# Stimuli - Transmission  -2.63127581 0.8124438 Inf  -3.239  0.0036
**
#
#P value adjustment: bonferroni method for 3 tests
#
#$`simple contrasts for Treatment`
#TreatmentGroup = Response:
# contrast    estimate        SE  df z.ratio p.value
# A - B    -1.76470276 0.8709916 Inf  -2.026  0.0428      *
#
#TreatmentGroup = Stimuli:
# contrast    estimate        SE  df z.ratio p.value
# A - B     0.05645295 0.7557887 Inf   0.075  0.9405
#
#TreatmentGroup = Transmission:
# contrast    estimate        SE  df z.ratio p.value
# A - B    -2.60217750 0.9684721 Inf  -2.687  0.0072    **


## To get the p-values for the loose variables!
n0<-
glmer(Flee~1+(1|ExpNr/GroupID),data=Maritafter,family=binomial(link="prob
it"),na.action=na.omit,control=glmerControl(calc.derivs=F))
n1<-update(n0,~.+Treatment)
n1a<-update(n0,~.+TreatmentGroup)
anova(n1,n0)
#Data: Maritafter
#Models:
#n0: Flee ~ 1 + (1 | ExpNr/GroupID)
#n1: Flee ~ (1 | ExpNr/GroupID) + Treatment
#   npar    AIC    BIC  logLik deviance  Chisq Df Pr(>Chisq)
#n0    3 237.61 248.05 -115.80   231.61
#n1    4 235.13 249.05 -113.56   227.13 4.4799  1     0.0343 *
```

```
#---
#Signif. codes:  0 '***' 0.001 '**' 0.01 '*' 0.05 '.' 0.1 ' ' 1

anova(n1a,n0)
#Data: Maritafter
#Models:
#n0: Flee ~ 1 + (1 | ExpNr/GroupID)
#n1a: Flee ~ (1 | ExpNr/GroupID) + TreatmentGroup
#    npar    AIC    BIC logLik deviance  Chisq Df Pr(>Chisq)
#n0     3 237.61 248.05 -115.8   231.61
#n1a    5 232.39 249.79 -111.2   222.39 9.2149  2   0.009977 **
#---
#Signif. codes:  0 '***' 0.001 '**' 0.01 '*' 0.05 '.' 0.1 ' ' 1


rm(m0a,m1a,m1b,n0,n1,n1a)

            ## Analyse time in dangerous zone!!
Marit$L1<-scale(Marit$Length,scale=FALSE,center=TRUE)

m0a<-
lme(Timedangerouszone~Treatment*BeforeOrAfter*TreatmentGroup+L1,random=~1
|ExpNr/GroupID/FishID,data=Marit,weights=varComb(varIdent(form=~1|BeforeO
rAfter),varIdent(form=~1|Treatment)),na.action=na.exclude)
summary(m0a)
#Linear mixed-effects model fit by REML
# Data: Marit
#       AIC      BIC    logLik
#  4917.176 4995.915 -2439.588
#
#Random effects:
# Formula: ~1 | ExpNr
#        (Intercept)
#StdDev:    15.36226
#
# Formula: ~1 | GroupID %in% ExpNr
#        (Intercept)
#StdDev:    18.90685
#
# Formula: ~1 | FishID %in% GroupID %in% ExpNr
#        (Intercept) Residual
#StdDev:    12.91075 28.19862
#
#Combination of variance functions:
# Structure: Different standard deviations per stratum
# Formula: ~1 | BeforeOrAfter
# Parameter estimates:
#  Before    After
#1.000000 1.272007
# Structure: Different standard deviations per stratum
# Formula: ~1 | Treatment
# Parameter estimates:
#       B        A
#1.000000 1.526687
#Fixed effects: Timedangerouszone ~ Treatment * BeforeOrAfter *
TreatmentGroup +      L1
#                                                                  Value
Std.Error  DF   t-value
```

```
#(Intercept)                                               203.04430
13.158642 223 15.430491
#TreatmentB                                                 57.60205
15.836874  18  3.637211
#BeforeOrAfterBefore                                        45.31644
12.313678 223  3.680171
#TreatmentGroupStimuli                                      16.79604
16.947247 184  0.991078
#TreatmentGroupTransmission                                  4.68311
16.948484 184  0.276314
#L1                                                         -1.26973
0.431146 184 -2.945000
#TreatmentB:BeforeOrAfterBefore                            -36.50441
14.040410 223 -2.599953
#TreatmentB:TreatmentGroupStimuli                          -20.45476
20.063943 184 -1.019479
#TreatmentB:TreatmentGroupTransmission                      10.23656
19.958088 184  0.512903
#BeforeOrAfterBefore:TreatmentGroupStimuli                  15.03984
17.414171 223  0.863655
#BeforeOrAfterBefore:TreatmentGroupTransmission             11.47959
17.414171 223  0.659209
#TreatmentB:BeforeOrAfterBefore:TreatmentGroupStimuli      -17.55349
19.849289 223 -0.884339
#TreatmentB:BeforeOrAfterBefore:TreatmentGroupTransmission -23.10422
19.851335 223 -1.163862
#                                                          p-value
#(Intercept)                                                0.0000
#TreatmentB                                                 0.0019
#BeforeOrAfterBefore                                        0.0003
#TreatmentGroupStimuli                                      0.3229
#TreatmentGroupTransmission                                 0.7826
#L1                                                         0.0036
#TreatmentB:BeforeOrAfterBefore                             0.0099
#TreatmentB:TreatmentGroupStimuli                           0.3093
#TreatmentB:TreatmentGroupTransmission                      0.6086
#BeforeOrAfterBefore:TreatmentGroupStimuli                  0.3887
#BeforeOrAfterBefore:TreatmentGroupTransmission             0.5104
#TreatmentB:BeforeOrAfterBefore:TreatmentGroupStimuli       0.3775
#TreatmentB:BeforeOrAfterBefore:TreatmentGroupTransmission  0.2457
# Correlation:
#                                                           (Intr) TrtmnB
BfrOAB TrtmGS TrtmGT
#TreatmentB                                                -0.831
#BeforeOrAfterBefore                                       -0.578  0.481
#TreatmentGroupStimuli                                     -0.644  0.535
0.449
#TreatmentGroupTransmission                                -0.644  0.535
0.449  0.500
#L1                                                        -0.017  0.004
0.000  0.004  0.013
#TreatmentB:BeforeOrAfterBefore                             0.507 -0.548
-0.877 -0.394 -0.394
#TreatmentB:TreatmentGroupStimuli                           0.544 -0.634
-0.379 -0.845 -0.422
#TreatmentB:TreatmentGroupTransmission                      0.547 -0.633
-0.381 -0.425 -0.849
```

```
#BeforeOrAfterBefore:TreatmentGroupStimuli                  0.409 -0.340
-0.707 -0.635 -0.318
#BeforeOrAfterBefore:TreatmentGroupTransmission             0.409 -0.340
-0.707 -0.318 -0.635
#TreatmentB:BeforeOrAfterBefore:TreatmentGroupStimuli      -0.359  0.388
0.620  0.557  0.279
#TreatmentB:BeforeOrAfterBefore:TreatmentGroupTransmission -0.359  0.385
0.620  0.279  0.557
#                                                          L1
TrB:BOAB TB:TGS TB:TGT
#TreatmentB
#BeforeOrAfterBefore
#TreatmentGroupStimuli
#TreatmentGroupTransmission
#L1
#TreatmentB:BeforeOrAfterBefore                             0.000
#TreatmentB:TreatmentGroupStimuli                           0.012  0.433
#TreatmentB:TreatmentGroupTransmission                      0.009  0.438
0.504
#BeforeOrAfterBefore:TreatmentGroupStimuli                  0.000  0.620
0.536  0.270
#BeforeOrAfterBefore:TreatmentGroupTransmission             0.000  0.620
0.268  0.539
#TreatmentB:BeforeOrAfterBefore:TreatmentGroupStimuli       0.000 -0.707
-0.610 -0.312
#TreatmentB:BeforeOrAfterBefore:TreatmentGroupTransmission  0.001 -0.709
-0.302 -0.612
#                                                          BOAB:TGS
BOAB:TGT TB:BOAB:TGS
#TreatmentB
#BeforeOrAfterBefore
#TreatmentGroupStimuli
#TreatmentGroupTransmission
#L1
#TreatmentB:BeforeOrAfterBefore
#TreatmentB:TreatmentGroupStimuli
#TreatmentB:TreatmentGroupTransmission
#BeforeOrAfterBefore:TreatmentGroupStimuli
#BeforeOrAfterBefore:TreatmentGroupTransmission             0.500
#TreatmentB:BeforeOrAfterBefore:TreatmentGroupStimuli      -0.877   -
0.439
#TreatmentB:BeforeOrAfterBefore:TreatmentGroupTransmission -0.439   -
0.877    0.501
#
#Standardized Within-Group Residuals:
#       Min         Q1        Med         Q3        Max
#-4.7068801 -0.3290654  0.1886937  0.5600389  2.5139864
#
#Number of Observations: 479
#Number of Groups:
#                         ExpNr             GroupID %in% ExpNr FishID
%in% GroupID %in% ExpNr
#                            20                              61
2

m1<-update(m0a,method="ML")
drop1(m1,test="C")
#Single term deletions
```

```
#
#Model:
#Timedangerouszone ~ Treatment * BeforeOrAfter * TreatmentGroup +
#    L1
#                                        Df    AIC    LRT Pr(>Chi)
#<none>                                     4991.5
#L1                                      1 4997.1 7.5474  0.00601 **
#Treatment:BeforeOrAfter:TreatmentGroup  2 4989.0 1.4952  0.47350
#---
#Signif. codes:  0 '***' 0.001 '**' 0.01 '*' 0.05 '.' 0.1 ' ' 1

m2<-update(m1,~.-Treatment:BeforeOrAfter:TreatmentGroup)
drop1(m2,test="C")
#Single term deletions
#
#Model:
#Timedangerouszone ~ Treatment + BeforeOrAfter + TreatmentGroup +
#    L1 + Treatment:BeforeOrAfter + Treatment:TreatmentGroup +
#    BeforeOrAfter:TreatmentGroup
#                              Df    AIC    LRT  Pr(>Chi)
#<none>                           4989.0
#L1                             1 4994.5  7.533  0.006058 **
#Treatment:BeforeOrAfter        1 5022.8 35.771 2.219e-09 ***
#Treatment:TreatmentGroup       2 4989.8  4.800  0.090702 .
#BeforeOrAfter:TreatmentGroup  2 4986.0  1.010  0.603375
#---
#Signif. codes:  0 '***' 0.001 '**' 0.01 '*' 0.05 '.' 0.1 ' ' 1

m3<-update(m2,~.-BeforeOrAfter:TreatmentGroup)
drop1(m3,test="C")
#Single term deletions
#
#Model:
#Timedangerouszone ~ Treatment + BeforeOrAfter + TreatmentGroup +
#    L1 + Treatment:BeforeOrAfter + Treatment:TreatmentGroup
#                          Df    AIC    LRT  Pr(>Chi)
#<none>                       4986.0
#L1                         1 4991.5  7.534  0.006053 **
#Treatment:BeforeOrAfter    1 5019.8 35.790 2.198e-09 ***
#Treatment:TreatmentGroup  2 4986.8  4.792  0.091076 .
#---
#Signif. codes:  0 '***' 0.001 '**' 0.01 '*' 0.05 '.' 0.1 ' ' 1

m4<-update(m3,~.-Treatment:TreatmentGroup)
drop1(m4,test="C")
#Single term deletions
#
#Model:
#Timedangerouszone ~ Treatment + BeforeOrAfter + TreatmentGroup +
#    L1 + Treatment:BeforeOrAfter
#                         Df    AIC    LRT Pr(>Chi)
#<none>                      4986.8
#TreatmentGroup           2 4984.4  1.598 0.449675
#L1                       1 4992.9  8.052 0.004545 **
#Treatment:BeforeOrAfter  1 5020.4 35.643 2.37e-09 ***
#---
#Signif. codes:  0 '***' 0.001 '**' 0.01 '*' 0.05 '.' 0.1 ' ' 1
```

```
m5<-update(m4,~.-TreatmentGroup)
drop1(m5,test="C")
#Single term deletions
#
#Model:
#Timedangerouszone ~ Treatment + BeforeOrAfter + L1 +
Treatment:BeforeOrAfter
#                         Df    AIC    LRT  Pr(>Chi)
#<none>                      4984.4
#L1                        1 4991.0  8.617  0.003329 **
#Treatment:BeforeOrAfter  1 5018.1 35.707 2.294e-09 ***
#---
#Signif. codes:  0 '***' 0.001 '**' 0.01 '*' 0.05 '.' 0.1 ' ' 1

m6<-update(m5,method="REML")
summary(m6)
#Linear mixed-effects model fit by REML
# Data: Marit
#       AIC      BIC    logLik
#  4962.818 5008.592 -2470.409
#
#Random effects:
# Formula: ~1 | ExpNr
#        (Intercept)
#StdDev:    15.10381
#
# Formula: ~1 | GroupID %in% ExpNr
#        (Intercept)
#StdDev:    19.72759
#
# Formula: ~1 | FishID %in% GroupID %in% ExpNr
#        (Intercept) Residual
#StdDev:    12.67253 28.33456
#
#Combination of variance functions:
# Structure: Different standard deviations per stratum
# Formula: ~1 | BeforeOrAfter
# Parameter estimates:
#  Before    After
#1.000000 1.263552
# Structure: Different standard deviations per stratum
# Formula: ~1 | Treatment
# Parameter estimates:
#       B        A
#1.000000 1.520982
#Fixed effects: Timedangerouszone ~ Treatment + BeforeOrAfter + L1 +
Treatment:BeforeOrAfter
#                                Value Std.Error  DF   t-value p-value
#(Intercept)                 210.21311  8.792495 227 23.908243  0.0000
#TreatmentB                   54.15222 10.817843  18  5.005824  0.0001
#BeforeOrAfterBefore          54.15625  7.087686 227  7.640892  0.0000
#L1                           -1.30135  0.430285 188 -3.024384  0.0028
#TreatmentB:BeforeOrAfterBefore -50.06706  8.067715 227 -6.205854  0.0000
# Correlation:
#                            (Intr) TrtmnB BfrOAB L1
#TreatmentB                  -0.813
#BeforeOrAfterBefore         -0.496  0.403
#L1                          -0.014  0.019  0.000
```

```
#TreatmentB:BeforeOrAfterBefore  0.435 -0.458 -0.879 -0.001
#
#Standardized Within-Group Residuals:
#       Min         Q1        Med         Q3        Max
#-4.7485921 -0.3609955  0.1901362  0.5443219  2.5872286
#
#Number of Observations: 479
#Number of Groups:
#                       ExpNr              GroupID %in% ExpNr FishID
%in% GroupID %in% ExpNr
#                          20                           61
250

qqPlot(resid(m6))
ad.test(resid(m6))

#        Anderson-Darling normality test
#
#data:  resid(m6)
#A = 11.383, p-value < 2.2e-16

plot(resid(m6,type="normalized")~Marit$Treatment)
leveneTest(resid(m6,type="normalized")~Marit$Treatment)
#Levene's Test for Homogeneity of Variance (center = median)
#       Df F value  Pr(>F)
#group   1  5.2188 0.02278 *
#      477
#---
#Signif. codes:  0 '***' 0.001 '**' 0.01 '*' 0.05 '.' 0.1 ' ' 1

plot(resid(m6,type="normalized")~Marit$BeforeOrAfter)
leveneTest(resid(m6,type="normalized")~Marit$BeforeOrAfter)
#Levene's Test for Homogeneity of Variance (center = median)
#       Df F value  Pr(>F)
#group   1   3.201 0.07423 .
#      477
#---
#Signif. codes:  0 '***' 0.001 '**' 0.01 '*' 0.05 '.' 0.1 ' ' 1



emmeans(m6,pairwise~Treatment|BeforeOrAfter,simple="each")$contrast
#$`simple contrasts for Treatment`
#BeforeOrAfter = After:
# contrast estimate   SE df t.ratio p.value
# A - B      -54.15 10.8 18 -5.006  0.0001     ***
#
#BeforeOrAfter = Before:
# contrast estimate   SE df t.ratio p.value
# A - B       -4.09 10.1 18 -0.404  0.6908
#
#Degrees-of-freedom method: containment
#
#$`simple contrasts for BeforeOrAfter`
#Treatment = A:
# contrast       estimate   SE  df t.ratio p.value
# After - Before   -54.16 7.09 227 -7.641  <.0001     ***
#
```

```
#Treatment = B:
# contrast       estimate   SE  df t.ratio p.value
# After - Before    -4.09 3.85 227 -1.061  0.2898
#
#Degrees-of-freedom method: containment



## Getting p-values of loose variables
n0<-
lme(Timedangerouszone~1,random=~1|ExpNr/GroupID/FishID,data=Marit,weights
=varComb(varIdent(form=~1|BeforeOrAfter),varIdent(form=~1|Treatment)),na.
action=na.exclude,method="ML")
n1a<-update(n0,~.+Treatment)
n1b<-update(n0,~.+BeforeOrAfter)
drop1(n1a,test="C")
#Single term deletions
#
#Model:
#Timedangerouszone ~ Treatment
#          Df    AIC    LRT Pr(>Chi)
#<none>       5037.3
#Treatment  1 5040.9 5.6359   0.0176 *
#---
#Signif. codes:  0 '***' 0.001 '**' 0.01 '*' 0.05 '.' 0.1 ' ' 1

drop1(n1b,test="C")
#Single term deletions
#
#Model:
#Timedangerouszone ~ BeforeOrAfter
#              Df    AIC    LRT Pr(>Chi)
#<none>           5028.3
#BeforeOrAfter  1 5040.9 14.612 0.000132 ***
#---
#Signif. codes:  0 '***' 0.001 '**' 0.01 '*' 0.05 '.' 0.1 ' ' 1




           ## Code for making and saving Fig. 2
# Setting font type
windowsFonts(Arial="TT Arial")

## Changing the names of the treatments!
levels(Maritafter$Treatment)[levels(Maritafter$Treatment)=="A"]<-
"Control"
levels(Maritafter$Treatment)[levels(Maritafter$Treatment)=="B"]<-
"Infected"

# Changing the orders of the treatment groups to a more logical order,
starting with the stimuli group that was exposed to the bird strike and
ending with the response group!
Maritafter$TreatmentGroup<-
factor(Maritafter$TreatmentGroup,levels=c("Stimuli","Transmission","Respo
nse"))
```

```
# Calculating the p-values for the flee zone analysis, to implement stars
in the graph!
stat.test <-
compare_means(Flee~TreatmentGroup,group.by="Treatment",data=Maritafter,me
thod="t.test")

## The R code for Fig. 2a and 2b (Flee zone)
d1r<-
ggplot(Maritafter,aes(x=TreatmentGroup,y=Flee))+facet_wrap(~Treatment)+ge
om_violin(trim=TRUE,scale="width",position="dodge",aes(fill=TreatmentGrou
p))+scale_fill_manual(values=c("#c6dbef", "#4292c6","#08519c"),labels=c("
stimulus  ","  transmission  ","  response"))+
geom_point(aes(x=TreatmentGroup),position=position_jitter(width=0.3,heigh
t=0),alpha=0.25,size=6)+
ylab("Escape zone")+xlab("Compartment
group")+theme(strip.text=element_text(size=rel(3.5)),strip.background=ele
ment_rect(colour="black",fill="transparent"))+stat_pvalue_manual(stat.tes
t,size=15,label.size=15,label="p.signif", hide.ns =
TRUE,y.position=c(1.06,1.15))+guides(fill=FALSE)+theme(plot.margin =
margin(10, 10, -2,
10,"mm"),axis.title.y=element_text(vjust=1,hjust=0.5),axis.title.x=elemen
t_blank(),axis.title=element_text(vjust=0,hjust=0.5,lineheight=1.2,colour
="black",size=rel(3.5)),axis.text.y=element_text(colour="black",size=rel(
3.5)),axis.text.x=element_blank(),panel.background=element_rect(colour="b
lack",fill="transparent"),plot.background=element_rect(fill="transparent"
,colour=NA),panel.grid.major=element_blank(),panel.grid.minor=element_bla
nk())+scale_y_continuous(breaks=c(0,1),labels=c("safe","dangerous"))+scal
e_x_discrete(labels=c("stimulus", "transmission","response","stimulus",
"transmission","response"))

# Calculating the p-values for the fleeing depth analysis, to implement
stars in the graph!
stat.test1 <-
compare_means(Fleeingdepth~TreatmentGroup,group.by="Treatment",data=Marit
after,method="t.test")

## The R code for Fig. 2c and 2d (Fleeoing depth)
d1k<-
ggplot(Maritafter,aes(x=TreatmentGroup,y=Fleeingdepth))+facet_wrap(~Treat
ment)+geom_boxplot(aes(x=TreatmentGroup,fill=TreatmentGroup),position=pos
ition_dodge(0.8),width=0.75,size=0.75)+scale_fill_manual(values=c("#c6dbe
f", "#4292c6","#08519c"),labels=c("  stimulus  ","  transmission  ","
response"))+stat_pvalue_manual(stat.test1,size=15,label.size=15,label="p.
signif", hide.ns = TRUE,y.position=c(0,5,-
5))+geom_jitter(aes(x=TreatmentGroup,group=TreatmentGroup),position=posit
ion_dodge(0.8),alpha=0.25,size=6)+ylab("Fleeing depth
(cm)")+xlab("Compartment
group")+theme(strip.text=element_blank(),strip.background=element_blank()
)+guides(fill=FALSE)+theme(plot.margin=margin(5, 5, 13,
5,"mm"),axis.title.y=element_text(vjust=1,hjust=0.5,margin=margin(r=10),s
ize=rel(3.5)),axis.title.x=element_blank(),axis.text.y=element_text(colou
r="black",size=rel(3.5)),axis.text.x=element_blank(),panel.background=ele
ment_rect(colour="black",fill="transparent"),plot.background=element_rect
(fill="transparent",colour=NA),panel.grid.major=element_blank(),panel.gri
d.minor=element_blank())+scale_x_discrete(labels=c("stimulus","transmissi
on","response","stimulus","transmission","response"))+scale_y_reverse(lim
its=c(60,-6))
```

```
# Calculating the n values for each box and setting the location of where
to add the n values in Fig. 2e and 2f
fun_length1 <- function(x){
  return(data.frame(y=-10,label= paste0("n=", length(x))))
}

## The R code for Fig. 2e and 2f (Fleeoing depth)
d1l<-
ggplot(Maritafter,aes(x=TreatmentGroup,y=Timedangerouszone))+facet_wrap(~
Treatment)+geom_boxplot(aes(x=TreatmentGroup,fill=TreatmentGroup),positio
n=position_dodge(0.8),width=0.75,size=0.75)+scale_fill_manual(values=c("#
c6dbef", "#4292c6","#08519c"),labels=c("  stimulus  ","  transmission
","
response"))+stat_summary(aes(x=TreatmentGroup,group=TreatmentGroup),posit
ion=position_dodge(0.8),fun.data=fun_length1,geom="text",size=9,col="blac
k")+geom_jitter(aes(x=TreatmentGroup,group=TreatmentGroup),position=posit
ion_dodge(0.8),alpha=0.25,size=6)+ylab("Time dangerous zone
(sec)")+xlab("Compartment
group")+theme(strip.text=element_blank(),strip.background=element_blank()
)+guides(fill=guide_legend(keywidth=3,keyheight=2))+theme(plot.margin=mar
gin(-9, 5, 5, 5,"mm"),axis.title.y=element_text(vjust=-
0.5,hjust=0.5,margin=margin(r=10),size=rel(3.5)),axis.title.x=element_tex
t(vjust=-
1,hjust=0.5,margin=margin(t=10),size=rel(3.5)),axis.text.y=element_text(c
olour="black",size=rel(3.5)),axis.text.x=element_text(colour="black",size
=rel(3.5)),panel.background=element_rect(colour="black",fill="transparent
"),plot.background=element_rect(fill="transparent",colour=NA),panel.grid.
major=element_blank(),panel.grid.minor=element_blank(),legend.background=
element_blank(),
legend.key=element_blank(),legend.position="bottom",legend.title=element_
blank(),legend.spacing.y=unit(2.0,'cm'),legend.spacing.x=unit(1.0,'cm'),l
egend.margin=margin(0.7,0.7,0.7,0.7,'cm'),legend.text=element_text(size=3
5,lineheight=3))+scale_x_discrete(labels=c("stimulus","transmission","res
ponse","stimulus","transmission","response"))+scale_y_continuous(expand=c
(0,0),limits=c(-20,330))


# Using ggarrange to combine the three graphs above to form Fig. 2a-2f.
figure <- ggarrange(d1r, d1k,d1l+ font("x.text", size = rel(3.5)),ncol =
1, nrow = 3,align = "v",common.legend = TRUE, legend = "bottom",
font.label = list(size = 50, color = "black", face = "bold"))
figure

# To save the graphs! The a), b) etc were added manually in Inkscape!
 ggsave(file='D:\\Users\\User\\Documents\\PhD Munster\\Supervision BSc
students\\BSc thesis 2018\\Marit\\Manuscript\\Submission proc b\\Revised
versions\\Revision2 stats\\combi graph bigger individual data
points1.jpeg',width=45,height=60,dpi=300,units="cm",bg="transparent")



                     ## Analysis behaviour versus parasiteload ###


              ## fleeing depth vs parasiteload
MaritInf<-subset(Maritafter,InfectionStatus=="Infected")
## Removed outliers after checking residuals
```

```
MaritInf1<-MaritInf[-c(8,38),]
MaritInf2<-MaritInf1[-c(7),]

n0a<-
lmer(Fleeingdepth~parasiteload+(1|GroupID),data=MaritInf2,na.action=na.ex
clude,control=lmerControl(calc.derivs=F))

summary(n0a)
#Linear mixed model fit by REML ['lmerMod']
#Formula: Fleeingdepth ~ parasiteload + (1 | GroupID)
#   Data: MaritInf2
#Control: lmerControl(calc.derivs = F)
#
#REML criterion at convergence: 303.4
#
#Scaled residuals:
#    Min      1Q  Median      3Q     Max
#-2.0134 -0.5085  0.1819  0.4589  2.2951
#
#Random effects:
# Groups   Name        Variance Std.Dev.
# GroupID  (Intercept)  0.00    0.000
# Residual             66.79    8.173
#Number of obs: 45, groups:  GroupID, 13
#
#Fixed effects:
#             Estimate Std. Error t value
#(Intercept)    39.960      7.356   5.432
#parasiteload  -74.642     38.644  -1.932
#
#Correlation of Fixed Effects:
#            (Intr)
#parasitelod -0.986


n1<-update(n0a,REML=F)
drop1(n1,test="C")
#Single term deletions
#
#Model:
#Fleeingdepth ~ parasiteload + (1 | GroupID)
#             Df    AIC    LRT Pr(Chi)
#<none>          322.73
#parasiteload  1 324.48 3.7442 0.05299 .
#---
#Signif. codes:  0 '***' 0.001 '**' 0.01 '*' 0.05 '.' 0.1 ' ' 1

anova(n2,n1)
#Data: MaritInf2
#Models:
#n2: Fleeingdepth ~ (1 | GroupID)
#n1: Fleeingdepth ~ parasiteload + (1 | GroupID)
#   Df    AIC    BIC  logLik deviance  Chisq Chi Df Pr(>Chisq)
#n2  3 324.48 329.90 -159.24   318.48
#n1  4 322.73 329.96 -157.37   314.73 3.7442      1    0.05299 .
#---
#Signif. codes:  0 '***' 0.001 '**' 0.01 '*' 0.05 '.' 0.1 ' ' 1
```

```
qqnorm(resid(n0a));qqline(resid(n0a))
ad.test(resid(n0a))
#
#        Anderson-Darling normality test
#
#data:  resid(n0a)
#A = 0.56715, p-value = 0.1337

shapiro.test(resid(n0a))
#
#        Shapiro-Wilk normality test
#
#data:  resid(n0a)
#W = 0.96957, p-value = 0.2792

plot(n0a)
plot(resid(n0a))



                 ## Time dangerous zone vs parasiteload
MaritInf<-subset(Marit,InfectionStatus=="Infected")
MaritInf1<-na.omit(MaritInf)
m0<-
lmer(Timedangerouszone~parasiteload*BeforeOrAfter+(1|GroupID/FishID),data
=MaritInf1,control=lmerControl(calc.derivs=F))
summary(m0)
#Linear mixed model fit by REML ['lmerMod']
#Formula: Timedangerouszone ~ parasiteload * BeforeOrAfter + (1 |
GroupID/FishID)
#   Data: MaritInf1
#
#REML criterion at convergence: 900.1
#
#Scaled residuals:
#    Min      1Q  Median      3Q     Max
#-4.0898 -0.0495  0.1962  0.3856  1.0993
#
#Random effects:
# Groups         Name        Variance Std.Dev.
# FishID:GroupID (Intercept)  170.2   13.04
# GroupID        (Intercept)  462.0   21.49
# Residual                   1197.5   34.60
#Number of obs: 92, groups:  FishID:GroupID, 49; GroupID, 13
#
#Fixed effects:
#                                 Estimate Std. Error t value
#(Intercept)                        216.33      29.52   7.328
#parasiteload                       319.34     153.97   2.074
#BeforeOrAfterAfter                  31.95      36.23   0.882
#parasiteload:BeforeOrAfterAfter  -162.21     192.71  -0.842
#
#Correlation of Fixed Effects:
#            (Intr) prstld BfrOAA
#parasitelod -0.961
#BfrOrAftrAf -0.611  0.611
#prstld:BOAA  0.596 -0.621 -0.979
```

```
m1<-update(m0,REML="F")
drop1(m1,test="C")
#Single term deletions
#
#Model:
#Timedangerouszone ~ parasiteload * BeforeOrAfter + (1 | GroupID/FishID)
#                            npar    AIC     LRT Pr(Chi)
#<none>                           949.37
#parasiteload:BeforeOrAfter    1 948.10 0.73189  0.3923

m2<-update(m1,~.-parasiteload:BeforeOrAfter)
drop1(m2,test="C")
#Single term deletions
#
#Model:
#Timedangerouszone ~ parasiteload + BeforeOrAfter + (1 | GroupID/FishID)
#              npar    AIC    LRT Pr(Chi)
#<none>             948.10
#parasiteload     1 949.78 3.6786 0.05512 .
#BeforeOrAfter    1 946.18 0.0856 0.76985
#---
#Signif. codes:  0 '***' 0.001 '**' 0.01 '*' 0.05 '.' 0.1 ' ' 1

m3<-update(m2,~.-BeforeOrAfter)
drop1(m3,test="C")
#Single term deletions
#
#Model:
#Timedangerouszone ~ parasiteload + (1 | GroupID/FishID)
#             npar    AIC    LRT Pr(Chi)
#<none>            946.18
#parasiteload    1 947.85 3.6715 0.05535 .
#---
#Signif. codes:  0 '***' 0.001 '**' 0.01 '*' 0.05 '.' 0.1 ' ' 1

m4<-update(m3,REML=T)
summary(m4)
#Linear mixed model fit by REML ['lmerMod']
#Formula: Timedangerouszone ~ parasiteload + (1 | GroupID/FishID)
#   Data: MaritInf1
#
#REML criterion at convergence: 919
#
#Scaled residuals:
#    Min      1Q  Median      3Q     Max
#-4.1918 -0.0531  0.1981  0.3647  1.0718
#
#Random effects:
# Groups         Name        Variance Std.Dev.
# FishID:GroupID (Intercept)  186.7   13.66
# GroupID        (Intercept)  460.1   21.45
# Residual                   1165.5   34.14
#Number of obs: 92, groups:  FishID:GroupID, 49; GroupID, 13
#
#Fixed effects:
#             Estimate Std. Error t value
#(Intercept)    232.30      23.35   9.947
```

```
#parasiteload   238.31     120.66   1.975
#
#Correlation of Fixed Effects:
#            (Intr)
#parasitelod -0.951

qqPlot(resid(m4))
ad.test(resid(m4))

#        Anderson-Darling normality test
#
#data:  resid(m4)
#A = 8.3544, p-value < 2.2e-16

plot(resid(m4,type="pearson")~fitted(m4))


          ## Graphs behaviour vs parasiteload ##

## The R code for Fig. 3a (Fleeing depth versus parasite load)
MaritInf<-subset(Maritafter,InfectionStat=="Infected")
MaritInf1<-MaritInf[-c(8,38),]
MaritInf2<-MaritInf1[-c(7),]

e1a<-
ggplot(MaritInf2,aes(x=parasiteload,y=Fleeingdepth))+geom_point(shape=17,
size=3)+scale_y_reverse(limits=c(50,0))+xlab("parasite
burden")+ylab("fleeing depth
(cm)")+guides(fill=guide_legend(keywidth=3,keyheight=2))+theme(plot.margi
n = margin(10, 10, 10,
10,"mm"),axis.title.y=element_text(margin=margin(r=10),hjust=0.5,colour="
black",family="Arial",size=rel(2.5),lineheight=1.2),axis.title.x=element_
text(margin=margin(t=10),hjust=0.5,size=rel(2.5),colour="black",family="A
rial",lineheight=1.2),axis.text=element_text(family="Arial",colour="black
",size=rel(2.25)),panel.border=element_rect(colour="black",fill=NA),panel
.background=element_rect(fill="white"))+xlim(c(0.10,0.25))+
geom_abline(slope=74.642,intercept=-39.960)

rm(MaritInf1,MaritInf2, MaritInf)

# The R code for Fig 3b (Time dangerous zone versus parasite load)
Marit$BeforeOrAfter<-
factor(Marit$BeforeOrAfter,levels=c("Before","After"))
MaritInf<-subset(Marit,InfectionStatus=="Infected")

e1b<-
ggplot(MaritInf,aes(x=parasiteload,y=Timedangerouszone,col=BeforeOrAfter)
)+geom_point(shape=17,size=3)+geom_abline(slope=319.34,intercept=216.33,c
ol="#000000")+geom_abline(slope=157.13,intercept=248.28,col="#bdbdbd")+sc
ale_colour_manual(values=c("#000000", "#bdbdbd"),labels=c(" before   ","
after   "))+scale_shape_manual(values=c(1,2),labels=c(" before   ","
after   "))+xlab("parasite burden")+ylab("time dangerous zone
(sec)")+guides(fill=guide_legend(keywidth=3,keyheight=2))+theme(plot.marg
in = margin(10, 10, 10,
10,"mm"),axis.title.y=element_text(margin=margin(r=10),hjust=0.5,colour="
black",family="Arial",size=rel(2.5),lineheight=1.2),axis.title.x=element_
text(margin=margin(t=10),hjust=0.5,size=rel(2.5),colour="black",family="A
rial",lineheight=1.2),axis.text=element_text(family="Arial",colour="black
```

```
",size=rel(2.25)),panel.border=element_rect(colour="black",fill=NA),panel
.background=element_rect(fill="white"),legend.background=element_blank(),
legend.key=element_blank(),legend.position=c(0.2,
0.075),legend.title=element_blank(),legend.text=element_text(family="Aria
l",size=28,lineheight=3))+ylim(c(150,310))


# To combine the two graph to form Fig. 3.
figure <- ggarrange(e1a, e1b,ncol = 2, nrow = 1,align = "h",font.label =
list(size = 14, color = "black", face = "bold"))
figure


## To save graph. The a) and b) were added manually in Inkscape!
ggsave(file='Location name\\Fleeing depht and TDZ vs parasiteload
corrected.png',width=40,height=20,dpi=300,units="cm",bg="transparent")
```
